# Prognostic Impact of the Increase in Cardiac Troponin Levels during Tafamidis Therapy in Patients with Transthyretin Cardiac Amyloidosis

**DOI:** 10.3390/jcm12144631

**Published:** 2023-07-12

**Authors:** Makiko Nakamura, Teruhiko Imamura, Ryuichi Ushijima, Koichiro Kinugawa

**Affiliations:** Second Department of Internal Medicine, University of Toyama, 2630 Sugitani, Toyama 930-0194, Japan; nakamura@med.u-toyama.ac.jp (M.N.);

**Keywords:** MGUS, infiltrative cardiomyopathy, electrical dyssynchrony, ATTR-CM

## Abstract

Background: Recent clinical trials have demonstrated that tafamidis (Pfizer Inc., New York, NY, USA) reduced all-cause mortality and the number of cardiovascular hospitalizations compared with placebo in patients with transthyretin cardiac amyloidosis. However, the optimal surrogate markers during tafamidis treatment remain unknown. Methods: Consecutive patients with transthyretin cardiac amyloidosis who received tafamidis in our institute between May 2019 and December 2022 were retrospectively evaluated. The prognostic impact of an increase in troponin I levels during tafamidis therapy was evaluated. Results: A total of 18 patients (median age 77 years, 84% male) were included. For 14-month tafamidis therapy on median, cardiac troponin I levels increased in five patients. The cumulative incidence of all-cause hospitalization was significantly higher in the troponin-increased group than in the others (100% versus 33%, *p* < 0.0001). Troponin increase was independently associated with the cumulative incidence of all-cause hospitalization with an adjusted hazard ratio of 5.14 (95% confidence interval 1.02–25.9, *p* = 0.048). Conclusions: The increase in cardiac troponin levels may be a reasonable surrogate marker of response to tafamidis therapy in patients with transthyretin cardiac amyloidosis.

## 1. Introduction

Amyloidosis is a group of diseases in which amyloid fibrils deposit in the extracellular spaces of different organs, ultimately leading to progressive multi-organ dysfunction [1]. As a result of gene mutations or as an aging-related phenomenon, transthyretin molecules may misfold and deposit in the heart and in other organs as amyloid fibrils [2]. Wild-type transthyretin cardiac amyloidosis (ATTR-CM) is a progressive disease that ultimately leads to death due to diastolic/systolic dysfunction, conduction disturbance, arrhythmias, and systemic dysfunction with comorbidity and advanced frailty among aged populations [3,4,5,6]. Wild-type ATTR-CM is sometimes underdiagnosed. Some patients are not diagnosed with ATTR-CM until advanced atrioventricular block or electrical dyssynchrony develops [7,8].

Tafamidis (Pfizer Inc., New York, NY, USA) binds to the thyroxine-binding site of transthyretin and stabilizes transthyretin tetramers [2]. In the ATTR-ACT trial, tafamidis reduced all-cause mortality and cardiovascular hospitalization compared with placebo [9]. However, several patients were excluded from this trial, such as those with a cardiac pacemaker [9]. Patients with New York Heart Association (NYHA) class III had a higher rate of cardiovascular-related hospitalizations compared with those with NYHA class I–II [9]. A beneficial effect of tafamidis on mortality was not observed until 18 months. Thus, optimal patient selection and careful monitoring during the therapy are of great importance for successful tafamidis therapy, although detailed therapeutic strategies remain unestablished.

Recently, high-sensitivity cardiac troponin T (hs-cTnT) was proposed as a practical marker to screen cardiac amyloidosis [10]. We hypothesized that an increase in cardiac troponin levels after the initiation of tafamidis may be a surrogate of unsuccessful tafamidis therapy. In this study, we investigated the prognostic impact of transition in cardiac troponin I levels after tafamidis initiation in patients with ATTR-CM.

## 2. Methods

### 2.1. Patient Selection

We retrospectively evaluated consecutive patients who started tafamidis therapy between May 2019 and December 2022 to treat their ATTR-CM, which was diagnosed by the presence of amyloid deposits in their endomyocardium. All patients met society’s requirements for the administration of tafamidis [1]. All patients initiated tafamidis 80 mg once daily. Patients were followed during tafamidis therapy for 18 months or until April 2023. All patients were followed during tafamidis therapy, which was defined as an observation period of this study (i.e., the observation was terminated when tafamidis was discontinued). This study was performed according to the Declaration of Helsinki. The use of patients’ anonymous clinical data were approved by the local institutional review board beforehand (IRB number, R2019166).

### 2.2. Clinical Management

All patients received standard medical therapy, including diuretics for chronic heart failure, in addition to tafamidis. The management of heart failure was conducted by board-certified cardiologists. Permanent pacemaker implantation was performed in patients with advanced atrioventricular block. If the patients met the recommendation criteria for implantable cardiovascular defibrillator and/or cardiac resynchronization therapy [11], these therapies were also performed.

Tafamidis was considered to be discontinued in several clinical scenarios after dedicated informed consent from patients and their relatives, including the requirement of non-pharmacological interventions, progression of frailty, and progression of heart failure [12].

### 2.3. Obtained Baseline Characteristics

Medical records of all patients at the time of administration of tafamidis were retrospectively reviewed. Data on baseline demographic characteristics were collected, including the numbers of previous heart failure hospitalizations, the duration of heart failure, NYHA classifications, and the prevalence of implantable cardiac pacemaker. The presence of a monoclonal gammopathy of undetermined significance (MGUS) was defined as an abnormal serum free-light-chain ratio and detection of a monoclonal protein on serum or urine immunofixation electrophoresis [13].

### 2.4. Measurements of Biomarkers

Plasma B-type natriuretic peptide (BNP), serum N-terminal pro-B-type natriuretic peptide (NT-proBNP), serum albumin, and serum creatinine levels were measured immediately before tafamidis administration. Serum levels of cardiac troponin I and hs-cTnT were measured within three months before tafamidis administration. Levels of cardiac troponin I were re-evaluated from 1 to 12 months after tafamidis administration. Plasma BNP and serum troponin I concentrations were measured using a commercially available assay (Abbott Japan, Matsudo, Japan). Serum NT-proBNP concentrations were measured by Elecsys NT-proBNP immunoassay (Roche Diagnostics Ltd., Rotkreuz, Switzerland). Hs-cTnT concentrations were measured using a commercially available assay (BML, Tokyo, Japan).

We divided all patients into 2 groups according to the increase/non-increase in cardiac troponin I levels from baseline to 1 to 12 months after administration of tafamidis.

### 2.5. Measurements of Other Clinical Data

Electrocardiographic data, including rhythm and QRS duration, were examined. Echocardiographic data, including interventricular septum thickness, left-ventricular ejection fraction, left-ventricle mass index, and the prevalence of aortic valve stenosis (>moderate) and tricuspid valve regurgitation (>moderate), were also investigated. The measurements of echocardiography were performed by physiological technicians and verified by echo cardiologists in a standard fashion as recommended by the American Society of Echocardiography’s guidelines [14,15]. The interventricular septum thickness was measured by M-mode in parasternal long-axis view. The left-ventricular ejection fraction was measured by the modified Simpson method in apical 4-chamber and 2-chamber views. The left-ventricle mass index was calculated by the linear method with the Devererux and Reichek cube formula. Concomitant medical therapy was also shown.

### 2.6. Statistical Assessments

Statistics were performed using JMP pro ver17.0 (SAS Institute Japan Ltd., Tokyo, Japan). Variables with *p* < 0.05 were considered significant. Continuous data were described as median and interquartile range and were compared between two groups using the Mann–Whitney U test. Categorical data were compared between two groups by Fischer’s exact test.

All cohorts were divided into two groups by the increase/non-increase in troponin levels during tafamidis therapy. Patients were followed during tafamidis therapy, which was defined as an observation period of this study. Patients were censored when they terminated tafamidis.

Cumulative incidence of all-cause hospitalization and worsening heart failure hospitalization were assessed using by the Kaplan–Meier method as primary outcomes. We calculated the event rate of total hospitalizations and heart failure hospitalizations per year during the tafamidis treatment period.

The impact of troponin increase on all-cause hospitalization was evaluated by Cox proportional-hazard ratio regression analyses. Several baseline characteristics were included in the univariable analyses as potential confounders, in addition to the troponin increase. Variables with *p* < 0.05 in the univariable analyses were included in the multivariable analyses.

## 3. Results

### 3.1. Baseline Demographics Data

The baseline demographics data of the enrolled 18 patients are shown in Table 1, including 17 wild-type and 1 variant type with Val30Met. The median age was 75 years old, and 15 of the participants (83%) were male. The baseline NYHA classifications were as follows: 12 patients with class II; 5 patients with class III; and 1 patient with class IV.

**Figure 1:**
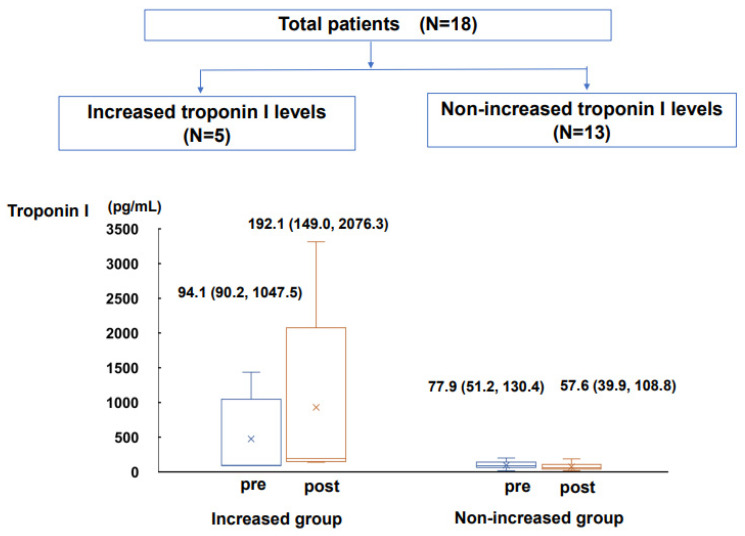
Changes in cardiac troponin I levels from pre-tafamidis treatment to post-tafamidis treatment in troponin-increased group and non-increased group were shown.

There were five patients with troponin increase during tafamidis therapy. In the other 13 patients, cardiac troponin I levels remained unchanged (Figure 1). The number of previous heart failure hospitalizations, heart failure duration, and NYHA classification were significantly higher in the troponin-increased group (*p* < 0.05 for all). There were three patients (17%) with concomitant MGUS (IgG-κ type), all of which were in the troponin-increased group.

### 3.2. Baseline Laboratory Data

The levels of BNP and NT-proBNP on median were 222 and 1898 pg/mL, respectively (Table 2). The levels of cardiac troponin I and hs-cTnT at baseline on median were 92.7 pg/mL and 0.053 ng/mL, respectively. There were no significant differences in baseline biomarkers between the two groups (*p* > 0.05 for all).

### 3.3. Other Baseline Data

The QRS duration was significantly longer in the troponin-increased group (154 versus 109 ms, *p* = 0.034; Table 3). Also, the prevalence of electrical dyssynchrony was significantly higher in the troponin-increased group (80% versus 23%, *p* = 0.047). There were no significant differences between the two groups in terms of baseline echocardiographic parameters and medication data (*p* > 0.05 for all; Table 3 and Table 4).

### 3.4. Tafamidis Therapy

Tafamidis therapy was continued for 14 (6.3, 22) months in the entire cohort (Table 5). Four patients terminated tafamidis until the end of study period given their refractoriness to tafamidis therapy. All of them were in the troponin-increased group. The dose of tafamidis was reduced in two patients in the troponin-non-increased group, due to headache and colitis, respectively.

### 3.5. Comparison in Clinical Outcomes

**Figure 2:**
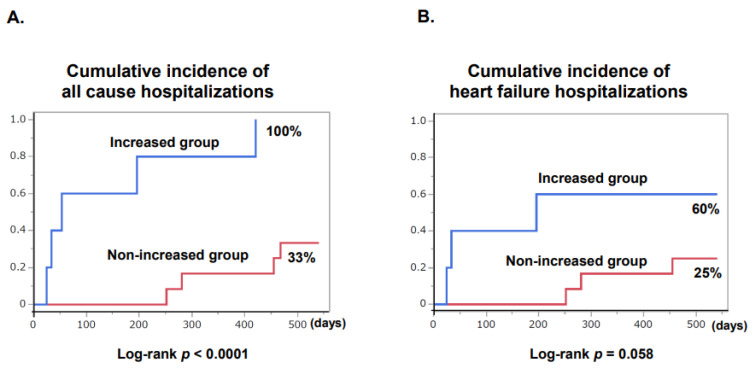
Cumulative incidence of all-cause hospitalization (**A**) and heart failure hospitalization (**B**) in troponin-increased group and non-increased group after initiation of tafamidis is shown.

No patients died during tafamidis therapy. The cumulative incidence of all-cause hospitalization was significantly higher in the troponin-increased group (100% versus 33%, *p* < 0.0001; Figure 2A). The cumulative incidence of heart failure hospitalization tended to be higher in the troponin-increased group (60% versus 25%, *p* = 0.058; Figure 2B).

Event rates of the troponin-increased group were significantly higher than those of the non-increased group in terms of all-cause hospitalization (event rate: 2.5 versus 0.22 events per year) and heart failure hospitalization (event rate: 0.81 versus 0.17 events per year).

In the multivariable analyses, troponin increase was independently associated with the cumulative incidence of the all-cause hospitalization with an adjusted hazard ratio of 5.14 (95% confidence interval 1.02–25.9, *p* = 0.048; Table 6).

## 4. Discussion

In this study, we investigated the association between the increase in cardiac troponin I levels and the clinical courses in patients with ATTR-CM receiving tafamidis therapy. An increase in troponin I levels during tafamidis therapy was independently associated with higher cumulative incidence of all-cause hospitalization.

### 4.1. Cardiac Amyloidosis and Electrical Dyssynchrony

Electrical dyssynchrony (QRS > 130 ms) in cardiac amyloidosis is associated with mortality and morbidity, as well as higher incidence of pacemaker implantation [8]. In our study, patients with a troponin increase had wider QRS duration but comparable prevalence of implanted pacemaker. Although it did not remain significant in the multivariable analysis, wide QRS (electrical dyssynchrony) itself may be a poor prognostic factor in this cohort, independent of pacemaker implantation [16].

### 4.2. Cardiac Troponin Level and Tafamidis Treatment

No studies evaluated the trend in hs-cTnT during tafamidis therapy, except for one study [6]. This study did not evaluate the trend in hs-cTnT levels in each patient. Given that the hs-cTnT is an important variable in the diagnosis of cardiac amyloidosis [10], it is not surprising that an increase in cardiac troponin I levels during tafamidis therapy was associated with a worse clinical course in our study. In patients who were refractory to tafamidis therapy, cardiac damage gradually progressed despite tafamidis therapy, and cardiac troponin levels, as a surrogate of myocardial damage, continuously increased.

### 4.3. Optimal Patient Selection

The current recommendation for optimal patient selection for tafamidis therapy is limited to NYHA class I or II. However, such a symptom-based classification is subjective. Furthermore, prognostic impacts of any objective markers, such as BNP level, have not been demonstrated thus far. In the previous study, baseline plasma BNP levels and estimated glomerular filtration rate were not associated with clinical outcomes [6].

In this study, we found that the increase in troponin levels was an independent predictor of worse prognosis, even after adjusting for baseline potential confounders, including baseline troponin levels, serum NT-proBNP levels, and QRS duration. We agree with the importance of baseline characteristics for risk stratification and optimal patient selection for tafamidis therapy, but according to our findings, a trend in troponin levels during tafamidis therapy may also have an additional prognostic impact.

Other markers may also be useful to follow during tafamidis therapy. However, Oche et al. demonstrated that several clinical parameters obtained during tafamidis therapy were not associated with future clinical outcomes.

According to our findings, we highly recommend following serum cardiac troponin levels to assess the efficacy of tafamidis, which may predict future clinical outcomes. Tafamidis therapy may be considered to be terminated in patients with persistently increased troponin levels despite tafamidis therapy, considering its cost effectiveness [12].

### 4.4. Study Limitations

This study was conducted retrospectively with a very small sample size at a single institute, and the observation period was just 14 months on median. One patient with the variant type was included in the study, and the clinical response of tafamidis may differ from those with the wild type, whereas the ATTR-ACT trial included both types and the clinical benefit of tafamidis was shown in both types.

There were no data on exercise capacity and quality of life. We have no global myocardial strain data in the echocardiography. We could not exclude the influence of concomitantly performed pharmacological and non-pharmacological therapies, other than tafamidis therapy.

## 5. Conclusions

An increase in cardiac troponin I levels during tafamidis therapy was independently associated with worse clinical outcomes in patients with ATTR-CM. An increase in troponin levels may be a practical surrogate biomarker to monitor and assess the clinical efficacy and predict future response to tafamidis therapy. Clinical implications of troponin-guided therapeutic strategies should be validated in the future study.

## Figures and Tables

**Table 1:** Baseline demographics data.

	Total (N = 18)	Troponin-Increased Group (N = 5)	Troponin-Non-Increased Group (N = 13)	*p*-Value
Age, years	75 (71, 79)	77 (73, 82)	72 (70, 78)	0.255
Sex: male, n (%)	15 (83%)	5 (100%)	10 (76.9%)	0.522
Wild type, n (%)	17 (94%)	5 (100%)	12 (92.3%)	1.000
Number of previous heart failure hospitalization	1 (1, 1.25)	2 (1, 4)	1 (1, 1)	0.018 *
Duration of heart failure (years)	1.1 (1.0, 3.2)	3.7 (1.4, 4.6)	1.0 (0.7, 2.2)	0.046 *
Body mass index (kg/m^2^)	23.0 (20.5, 24.4)	24.3 (20.2, 28.7)	22.6 (20.4, 23.9)	0.218
NYHA functional classification				0.001 *
class II	12 (67%)	0 (0%)	12 (92%)	
class III	5 (2.8%)	4 (80%)	1 (7.7%)	
class IV	1 (5.6%)	1 (20%)	0 (0%)	
IgG-κ type MGUS, n (%)	3 (17%)	3 (60%)	0 (0%)	0.002 *
Atrial fibrillation, n (%)	6 (33%)	1 (20%)	5 (38%)	0.615
Cardiac pacemaker, n (%)	6 (33%)	2 (40%)	4 (31%)	1.000
ICD/CRTD, n (%)	2 (11%)	2 (40%)	0 (0%)	0.065

NYHA, New York Heart Association; MGUS, monoclonal gammopathy of undetermined significance; ICD, implantable cardiovascular defibrillator; CRTD, cardiac resynchronization therapy with cardioverter-defibrillator. * *p* < 0.05 by Mann–Whitney U test or Fisher’s exact test as appropriate.

**Table 2:** Baseline laboratory data.

	Total (N = 18)	Troponin-Increased Group (N = 5)	Troponin-Non-Increased Group (N = 13)	*p*-Value
Plasma B-type natriuretic peptide (pg/mL)	222 (112, 418)	349 (187, 588)	207 (87, 250)	0.193
Serum N-terminal pro-B-type natriuretic peptide (pg/mL)	1898 (962, 3524)	3680 (2802, 6155)	1355 (802, 3135)	0.091
eGFR (mL/min/1.73 m^2^)	51.6 (39.2, 59.3)	44.3 (33.8, 56.9)	51.7 (43.2, 61.2)	0.257
Serum creatinine (mg/dL)	1.12 (0.94, 1.26)	1.26 (0.98, 1.58)	1.05 (0.89, 1.22)	0.199
Serum albumin (mg/dL)	4.0 (3.8, 4.2)	4.0 (3.6, 4.2)	4.0 (3.8, 4.2)	0.514
Troponin-I (pg/mL)	92.7 (65.7, 150.2)	192.1 (91.7, 1047.5)	95.0 (51.2, 124.8)	0.104
Hs-cTnT (ng/mL)	0.053 (0.030, 0.078)	0.078 (0.064, 0.130)	0.048 (0.029, 0.065)	0.058
Free-light-chain ratio (κ/λ)	1.40 (1.25, 1.80)	1.95 (1.27, 2.28)	1.37 (1.23, 1.66)	0.127

Hs-cTnT, high-sensitivity cardiac troponin T.

**Table 3:** Baseline electrocardiographic and echocardiographic data.

	Total (N = 18)	Troponin-Increased Group (N = 5)	Troponin -Non-Increased Group (N = 13)	*p*-Value
Electrocardiographic data				
QRS duration (ms)	113 (102, 155)	154 (126, 194)	109 (94, 135)	0.034 *
QRS duration (>130 ms), n (%)	7 (39%)	4 (80%)	3 (23%)	0.047 *
Echocardiographic data				
Interventricular septum thickness (mm)	14 (13, 17)	14.0 (13.5, 16.3)	13.0 (12.0, 15.0)	0.251
Left-ventricular mass index (g/m^2^)	172 (151, 221)	162 (146, 260)	177 (146, 227)	0.883
Left-ventricular end-diastolic diameter (mm)	45 (41, 50)	40 (38, 51)	45 (43, 50)	0.489
Left-ventricular ejection fraction (%)	52 (46, 58)	40 (27, 57)	52 (51, 59)	0.167
Aortic valve stenosis (>moderate)	1 (5.6%)	1 (20%)	0 (0%)	0.278
Tricuspid valve regurgitation (>moderate)	2 (11%)	1 (20%)	1 (7.7%)	0.490

* *p* < 0.05 by Mann–Whitney U test or Fisher’s exact test as appropriate.

**Table 4:** Baseline medication data.

	Total (N = 18)	Troponin-Increased Group (N = 5)	Troponin -Non-Increased Group (N = 13)	*p*-Value
ACE inhibitor/ARB, n (%)	13 (72%)	2 (40%)	11 (85%)	0.099
Dose of beta blockers, carvedilol equivalent (mg)	1.875 (0, 8.125)	0 (0, 6.25)	2.5 (0, 10)	0.468
Mineralocorticoid receptor antagonist, n (%)	12 (67%)	4 (80%)	8 (62%)	0.615
Dose of loop diuretics, furosemide equivalent (mg)	20 (7.5, 40)	40 (5, 60)	20 (5, 30)	0.420
Dose of tolvaptan (mg)	1.88 (0, 4.69)	3.75 (1.875, 11.25)	0 (0, 3.75)	0.098
Antiplatelet therapy, n (%)	2 (11%)	0 (0%)	2 (15%)	1.000
Anticoagulant therapy, n (%)	7 (39%)	2 (40%)	5 (38%)	1.000

ACE, angiotensin-converting enzyme; ARB, angiotensin receptor blocker.

**Table 5:** Clinical course during tafamidis therapy.

	Troponin-Increased Group (N = 5)	Troponin -Non-Increased Group (N = 13)	*p*-Value
Discontinuation of tafamidis	4 (80%)	0 (0%)	0.0016 *
Dose reduction of tafamidis	0 (0%)	2 (15%)	1.0000
Total tafamidis administration period (months)	8.2 (3.9, 18)	18 (8.2, 24)	0.1833
Clinical endpoints			
Death	0 (0%)	0 (0%)	
Heart failure hospitalization	3 (60%)	3 (23%)	0.2682
Non-cardiovascular hospitalization	2 (40%)	1 (7.7%)	0.1716

Continuous variables are presented as median and interquartile. * *p* < 0.05 by Mann–Whitney U test.

**Table 6:** Univariable and multivariable analyses for all-cause hospitalization.

	Univariable Analyses		Multivariable Analyses	
	Hazard Ratio (95% CI)	*p*-Value	Hazard Ratio (95% CI)	*p*-Value
Age (years old)	0.99 (0.91–1.08)	0.783		
Male	0.86 (0.18–4.03)	0.849		
Baseline log NT-proBNP (pg/mL)	4.45 (0.63–34.92)	0.135		
Baseline troponin-I (pg/dL)	1.00 (0.99–1.01)	0.259		
Baseline QRS duration (ms)	1.03 (1.01–1.06)	0.003 *	1.02 (0.99–1.05)	0.0648
Troponin increase	10.53 (2.41–46.13)	0.002 *	5.14 (1.02–25.91)	0.0475 *

NT-proBNP, N-terminal pro-B-type natriuretic peptide. CI, confidence interval. * *p* < 0.05 by logistic regression analysis.

## Data Availability

Data are available from the corresponding author upon reasonable request.
